# Nutritional Profile, Techno‐Functional, and Thermal Properties of Wheat Flour Enriched With Yellow Mopane Worm (*Gonimbrasia belina*) Powder

**DOI:** 10.1002/fsn3.72225

**Published:** 2026-08-11

**Authors:** Tina Sinethemba Bebe, Vusi Vincent Mshayisa, Lusani Norah Vhangani, Mpho Edward Mashau

**Affiliations:** ^1^ Department of Food Science and Technology Cape Peninsula University of Technology Bellville Cape Town South Africa; ^2^ Department of Food Science and Technology, Faculty of Science, Engineering and Agriculture University of Venda Thohoyandou South Africa

**Keywords:** amino acids, composite flours, FT‐IR spectroscopy, *Gonimbrasia belina*, minerals composition, yellow mopane worms

## Abstract

Mopane worms (*Gonimbrasia belina*) are a good source of key nutrients and have made significant contributions to the diets of sub‐Saharan African communities. The aim of this study was to evaluate yellow mopane worm powder (MWF) as a functional ingredient in wheat‐based composite flours by characterizing the nutritional, physicochemical, techno‐functional, pasting, and thermal properties of wheat flour (WF), MWF, and composite flours containing 5%, 10%, 15%, and 20% MWF substitution. Protein, ash, and fat contents varied across samples, with ranges of 14.99%–43.39%, 1.05%–7.97%, and 0.18%–5.22%, respectively. While moisture and energy content depicted no significant differences (*p* > 0.05). Enrichment with MWF enhanced the mineral profile of the composite flours, particularly for Na, K, P, Zn, and Fe, and increased essential amino acid content, including lysine (0.58–1.55 g/100 g), a key limiting nutrient in cereal‐based diets. Color analysis revealed significant increases (*p* < 0.05) in lightness, redness, and yellowness with higher MWF incorporation. Bulk density (0.57–0.76 g/mL), water activity (0.56–0.63), and pH (5.77–6.12) remained within acceptable ranges. MW15% and MW20% demonstrated significantly improved oil‐binding capacity and foaming stability, while MWF exhibited the highest water‐binding capacity. Conversely, foaming capacity, emulsifying capacity, and emulsifying stability decreased with increasing MWF content. Pasting properties indicated that MW5%, MW10%, and MW15% had peak, trough, and set viscosity profiles comparable to those of WF, suggesting their suitability for dough‐based applications. Thermal analysis (TGA and DSC) revealed strong thermal stability in MWF, with both endothermic and exothermic transitions observed across all samples. FT‐IR spectroscopy confirmed prominent amide I and II bands in high‐protein MWF, contrasting with weaker protein‐associated absorption in WF.

## Graphical abstract

Yellow mopane worm powder enhanced the nutritional and functional properties of wheat‐based composite flours by increasing protein, lysine, and mineral contents. Substitution levels up to 15% maintained pasting characteristics comparable to wheat flour, highlighting its suitability for the development of nutrient‐dense cereal products.

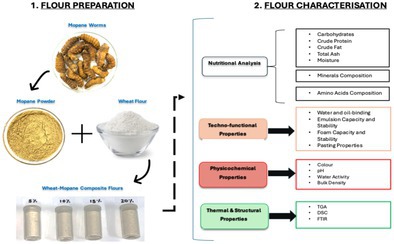

## 1 Introduction

In recent years, there has been a growing interest in alternative protein sources that are more sustainable, cost‐effective, and environmentally friendly. Consequently, this has led to the emergence of the concept of entomophagy in academic literature (Willeke et al. 2025). With the impending challenge of the world's population reaching 8.6 billion by 2030 and 9.8 billion by 2050 (UN 2022), edible insects have attracted attention as a novel, reliable source of nutrition, offering an opportunity to address global concerns about food insecurity and malnutrition. In many developing economies, edible insects remain an essential traditional food source and are estimated to be consumed by approximately 2 billion people worldwide (Van Huis 2013). Due to the nutritional profile and the sustainability and efficiency of edible insect rearing, the Food and Agriculture Organization (FAO) of the United Nations (UN) issued a call in 2013 for countries to promote entomophagy (FAO 2024). Consequently, researchers have explored various approaches to integrating edible insects into food systems as functional ingredients in disguised or less recognizable forms, since food neophobia continues to limit consumer acceptance (Vanqa et al. 2022).

Wheat flour remains the dominant flour for bread, pasta, and baked products globally, yet its nutritional value is limited by rapidly digestible starch, which contributes to an increased glycaemic index. Nevertheless, wheat flour is rich in carbohydrates (68%–88%), has a moderate protein content, and contains low levels of vitamins and minerals (Orkusz and Orkusz 2025). Significant amounts of minerals are lost during milling, including 90% of manganese, 80% of magnesium, potassium, and copper, and 85% of zinc, compared to whole wheat flour (Heshe et al. 2015). Furthermore, vitamins are destroyed by heat during milling, resulting in low levels in wheat flour. Milling also removes the wheat flour fiber, resulting in a low fiber content of 0.51 g/100 g (Mihaly Cozmuta et al. 2023). Wheat flour is deficient in some essential amino acids, especially lysine, which is the limiting amino acid in cereal flours (Tanga et al. 2025). The incorporation of edible insect flour into wheat flour may offer targeted solutions since insects are rich in amino acids and minerals. The most substantial enhancement arises from addressing wheat's lysine deficiency, as insect species have higher essential amino acids that are underrepresented in wheat (Carcea 2020). Furthermore, insect flour provides significant mineral enrichment, unlike wheat flour, which loses 80%–90% of key minerals during milling (Mihaly Cozmuta et al. 2023). The incorporation of mealworm flour increased the potassium, phosphorus, copper, and zinc content of wheat crackers by 15% (Djouadi et al. 2022).

One of the most popular edible insects, especially in Southern Africa, is *Gonimbrasia belina* (*G. belina*), also known as the mopane worm or mopane caterpillar. The mopane worm is known to be primarily found on the mopane tree ( *Colophospermum mopane* ), from which the caterpillar derives its nutrition (Kwiri et al. 2020). However, it has also been reported to obtain nutrition from trees of other species (Nsevolo et al. 2023). Nutritionally, mopane worms offer significant amounts of protein, fat, and carbohydrate content, as well as substantial levels of minerals, such as calcium and phosphorus, and amino acids, including lysine, tryptophan, and methionine, along with high levels of vitamins (Mnisi et al. 2022; Ruzengwe et al. 2023). While the vast nutritional potential of *G. belina* has been reported in the literature, its performance as a functional ingredient for fortifying nutrient‐deficient foods must still be investigated and better understood to enable the edible insect to be incorporated into familiar foods. Key indicators that provide insight into the food's potential use as a functional ingredient include its techno‐functional properties, physical properties, thermal and structural behavior.

An ingredient's techno‐functionality can be defined as any property of a food, besides its nutritive value, that impacts its utilization and performance (Kostić et al. 2015; Mshayisa et al. 2022) and is critical for the acceptability and sensorial properties of a finished product. Solubility, emulsification, gelation, foaming properties, water‐holding capacity, and oil absorption capacity are among the techno‐functional properties that are vital during food processing. Additionally, to further promote the application of insect‐derived functional ingredients, their structural and thermal behavior, which influences the techno‐functional properties, must be studied (Mshayisa et al. 2021). To date, and to the best of our knowledge, this study is among the first conducted in the Global South to provide an in‐depth characterization of the yellow mopane worm by examining its nutritional, physicochemical, techno‐functional, structural, and thermal properties, with the view of utilizing it as a functional food ingredient. Mshayisa et al. (2025) evaluated the effect of black mopane worm powder on the nutritional, techno‐functional, and structural properties of wheat flour. Their findings showed that the protein, ash, and fat content of wheat flour increased with increasing mopane worm substitution levels. The addition of mopane worm powder decreased the lightness of the wheat flour, while the redness increased with the substitution levels. Although several studies have established mopane worms as nutrient‐dense ingredients (Mashau et al. 2026; Vanqa et al. 2022) with considerable potential for food applications (Mshayisa et al. 2025), existing research has predominantly focused on the black mopane worms. Consequently, yellow mopane worms remain underexplored, particularly with respect to their functionality and performance in food systems compared with black mopane worms. Therefore, the objective of this study was to establish the nutritional composition (including amino acids and minerals), physicochemical, techno‐functional, and structural and thermal properties of composite flours comprising wheat flour and yellow mopane worm powder. The study focused on the yellow mopane worm variety to provide a standardized basis for evaluating its nutritional and techno‐functional suitability in baked products, as color variations among mopane worms may contribute to compositional variability.

## 2 Materials and Methods

### 2.1 Source of Materials

Air‐dried yellow mopane worms were sourced from informal street vendors in Thohoyandou, Limpopo province, in South Africa. All chemicals used in the study were sourced from Merck (Sigma‐Aldrich, Johannesburg, South Africa) unless otherwise stated and were of analytical grade. The water used in the study was purified using the Milli‐Q purification system (Millipore, Microsep, Bellville, South Africa). Equipment used in the study was obtained from the Departments of Food Science and Technology and Clothing & Textiles.

### 2.2 Preparation of Mopane Worm (*G. belina*) Powder and Mopane‐Wheat Composite Flours

Mopane worms were ground using a laboratory grinder (Kenwood Power Blender BLM45.240SS, United Kingdom) and thereafter passed through a 500 μm sieve to obtain the mopane worm powder. The powder was stored in zip‐lock bags and in an airtight container until it was ready for use. The coarse powder not passed through the sieve was discarded.

In preparation for the composite flour blends, yellow mopane worm powder (MWF) and wheat flour (WF) were weighed and mixed at different ratios, with MWF replacing WF at concentrations of 5%, 10%, 15%, and 20% (w/w). The blends were then coded as MW5%, MW10%, MW15% and MW20% respectively. Once blended, the composite flours were stored at room temperature in polyethene bags and airtight containers until analysis. In this study, 100% WF (WF) and 100% MWF (MWF) were used as control samples.

### 2.3 Proximate Composition Analysis

Proximate composition, namely crude protein (920.87), crude fat (932.06), moisture (925.10), and total ash (923.03) of the wheat flour, composite flours, and mopane worm powder were analyzed following methods recommended by the Association of Official Analytical Chemists (AOAC International 1990). Crude protein was analyzed using the Dumas method (TruSpec Leco Carbon/Hydrogen/Nitrogen Series, Leco Africa), with EDTA as the standard. The nitrogen‐to‐protein conversion factor of 5.60, as recommended by Janssen et al. (2017), was used to determine the crude protein content. Crude fat was determined using a Soxhlet extraction machine (ST 243 Soxhlet 1040 extraction unit) with petroleum ether heated to 90°C for 30 min. The solvent was then evaporated, and the remaining fat in the extraction cups was weighed. Moisture content was determined by drying samples at 98°C–100°C in a vacuum oven for 3 h, then reweighing to determine the change in moisture content, expressed as a percentage. The total ash was determined by first charring the weighed sample over an open flame and then placing the charred sample in a muffle furnace at 550°C for 12 h to ash. Once cooled, the ashed sample was weighed. The percentage carbohydrate content was determined by difference on a dry basis by subtracting all components (crude protein, crude fat, moisture, and total ash) from 100. The energy value, expressed in calories, was determined using the Atwater formula, as recommended by the FAO:

$$\begin{array}{c} \text{Energy}\;\left(\text{kCal}/100g\right)=\\ 4\times \left(\text{Carbohydrates}\;\left(g\right)+\text{Protein}\;\left(g\right)\right)+9\times \mathrm{Fat}\;\left(g\right) \end{array}$$

### 2.4 Mineral Composition Analysis

The mineral composition analysis was conducted following the methods described by Queiroz et al. (2021). Approximately 5 g of the sample was prepared and ashed at 500°C for 5 h, then cooled to ambient temperature. Thereafter, 5 mL of 1 M nitric acid was added to the sample, which was then filtered into a 100 mL volumetric flask, and additional nitric acid was added to fill the solution to volume. An Inductively Coupled Plasma‐Mass Spectrometer (ICP‐MS) was used to measure the elemental composition of the composite flours and baked goods.

### 2.5 Amino Acid Analysis

Following the method adopted from Mshayisa et al. (2022), the amino acid composition of the composite flours was analyzed. Firstly, approximately 0.5 g of the respective composite flour samples was hydrolyzed using 6 mL of 6 M HCl at 110°C for 23 h. Subsequently, the internal standard, which was prepared by adding 7.5 mL of 5 nM norleucine in water, was added to the samples. After undergoing derivatization with 6‐aminoquinolyl‐N‐hydroxysuccinimidyl carbamate (AQC), the samples were analyzed using the High‐Performance Liquid Chromatography with a fluorescence detector (HPLC‐FLD) (HPLC/FLD, Waters Alliance 2695, Agilent Technologies, Chemetrix, Midrand, South Africa).

### 2.6 Determination of the Physicochemical Properties in Composite Flours and Baked Products

#### 2.6.1 Color

The color of the composite flours was determined following Diedericks and Jideani (2015), with some modifications. The samples were decanted into a small 30 mm glass sample holder, ensuring that the bottom was evenly covered to allow reflectance measurement. The color was measured in triplicate using a spectrophotometer (Konica Minolta Sensing Americas Inc., Tokyo, Japan), which was first calibrated using a white calibration plate and then a black calibration plate (zero) prior to measuring the flour samples. The CIEL* a* b* color space was used to interpret the results. The average of three color readings taken at various positions (represented by one reading on the machine) was obtained for L* (lightness), a* (+a* = red and −a* = green) and b* (+b* = yellow and −b* = blue). Color differences between the flours were calculated using the color difference equation:

$$∆E=\sqrt{∆L^{*2}+∆a^{*2}+∆b^{*2}}$$

#### 2.6.2 Bulk Density

Following the method outlined by Vanqa et al. (2022), the bulk density of the composite flours was determined using a measuring cylinder (V), which was first tared on the scale. Subsequently, the cylinder was filled with the sample, lightly tapped to ensure that the flour had settled properly, and refilled again, as required. Once filled to the mark, the cylinder was weighed to obtain the mass (M) of the flour in the cylinder, and bulk density was determined by dividing the mass obtained by the volume of the cylinder (V) and expressing the results in g/mL:

$$\text{Bulk density}\;\left(\frac{g}{\mathrm{mL}}\right)=\frac{M}{V}$$

#### 2.6.3 Water Activity (Aw) and pH

The water activity of the composite flours was determined using a water activity meter (Novasina MS1 Set‐AW, Lachen, Switzerland). Prior to testing, the meter's accuracy was verified using three different salt‐humidity standards (SAL‐T 33%, SAL‐T 75%, and SAL‐T 90%). Thereafter, approximately 3 g of the sample was decanted into a sample dish and placed inside the device measurement cell. The A_w_ was measured in triplicate for 80 s, and the readings for each sample were recorded. To measure the pH of the composite flours, solutions at 5%, 10%, 15%, and 20% flour concentration were prepared in deionized water in a 50 mL glass beaker. The measurement was then conducted at 25°C using a pH meter. Prior to use, the device was calibrated with standard buffers at pH 4, 7, and 10 to ensure precise results.

### 2.7 Determination of Techno‐Functional Properties of Composite Flours

For this study, the techno‐functional properties evaluated on the insect‐derived composite flours included water and oil‐binding capacity, emulsion capacity and stability, foaming capacity and stability, and pasting properties.

#### 2.7.1 Water and Oil‐Binding Capacity

The water‐binding capacity (WBC) and oil‐binding capacity (OBC) were determined according to the methods of Purschke et al. (2018) and Mshayisa et al. (2022), with minor modifications. Approximately 0.5 g of the various composite flour samples was mixed with 2.5 mL deionized water, vortexed (Vortex‐Genie 2, Scientific Industry Inc., Bohemia, NY, USA) for 60 s and then centrifuged (Thermo Electron Corporation Jouan MR1812, Waltham, MA, USA) at room temperature for 20 min at 3220 × g, respectively. The supernatant was decanted, and the centrifuge tube containing the sample was placed upside‐down on filter paper for 60 min, then weighed. The WBC was determined by calculating the difference between the final weight and the initial weight and dividing by the dried weight of the sample:

$$\mathrm{WBC}\;\left(\frac{g}{g}\right)=\frac{W_{b}-W_{a}}{W_{a,\mathrm{DM}}}$$

where W_a_ is the initial weight, W_b_ is the final weight, W_a,DM_ is the weight based on dry matter.

The OBC of the samples was determined using the same methodology as for the WBC, except that commercial sunflower oil was used in place of deionized water, and the vortexing step was performed for 120 s rather than 60 s.

#### 2.7.2 Emulsion Capacity and Stability

The emulsion capacity (EC) and emulsion stability (ES) were determined according to the methods outlined by Vanqa et al. (2022) with minor revisions. Solutions (5% w/v) were prepared by dispersing the various composite flour samples in distilled water and homogenized (Polytron PT 2500 E, Thermofisher, Cape Town, South Africa) at 18,000 rpm for 1 min. Aliquots (15 mL) of the respective samples were then mixed with commercial sunflower oil (1:1 v/v) and homogenized at 10,000 rpm for 3 min. Thereafter, the samples were centrifuged at 3220 × g for 5 min, and the volumes of the individual layers were measured using a 50 mL graduated cylinder. The EC was calculated using the equation presented below:

$$\mathrm{EC}\;\left(\%\right)=\frac{V_{\mathrm{el}}}{V}\times 100$$

Subsequently, the ES was determined by heating the emulsion in a water bath of 80°C for 30 min, and the following equation was used to determine the stability value:

$$\mathrm{ES}\;\left(\%\right)=\frac{V_{30}}{V_{\mathrm{el}}}\times 100$$

where V = total volume of the tube contents, V_el_ = volume of emulsified layer, V_30_ = volume of emulsified layer after heating for 30 min.

#### 2.7.3 Foaming Capacity and Stability

The foaming capacity (FC) and stability (FS) of the composite flour were determined following the methods adapted from Vanqa et al. (2022) and Mshayisa et al. (2022), respectively. First, a solution (5% w/v) was made by dispersing the composite flour samples in deionized water. Aliquots (20 mL) of the solution were then homogenized in a high‐shear homogenizer mixer (Polytron PT 2500E, United Scientific, Cape Town, South Africa) at 16,000 rpm for 3 min and poured into a 50 mL graduated cylinder. The total volume was read at time zero and then 30 min after homogenization. The FC and FS were determined using the following equations, and the results were expressed as a percentage:

$$\mathrm{FC}\;\left(\%\right)=\frac{V_{a}-V}{V}\times 100$$

and

$$\mathrm{FS}\;\left(\%\right)=\frac{V_{30}}{V_{a}}\times 100$$

where V = volume before whipping, V_a_ = volume after whipping, V_30_ = volume 30 min after homogenization.

#### 2.7.4 Pasting Properties of the Composite Flours

The rheological properties of the composite flours and control samples were evaluated using a Rapid Visco Analyzer (RVA) (Model RVA‐4500, PerkinElmer (Perten), Australia). The first step was to determine the moisture percentage of the flour samples using the moisture analyzer. The moisture result was then entered into the RVA software programme, which helped calculate the sample weight and the water required for the analysis. Based on the moisture readings for all samples, which ranged between 8% and 11%, 3 g of flour and 25 g of water were calculated for analysis. The water and flour were then weighed and decanted into aluminium cups, respectively. Using the spindle on the RVA machine, the flour and water were agitated to break up any large flour particles and form a slurry. Thereafter, the aluminium cup was affixed to the RVA machine using the spindle. While stirring at 160 rpm, the samples were heated to 50°C for 1 min, then the temperature increased to 95°C and was held for 2.5 min. The samples were then finally cooled back to 50°C. At the end of the machine cycle, the following values relating to the pasting properties were obtained: pasting temperature, which is the temperature the starch granules start swelling; breakdown viscosity, which is the difference between the minimum and maximum viscosity during heating; final viscosity, which is the viscosity at the end of cooling period; setback viscosity, which is the difference between final viscosity and the minimum viscosity during cooling.

### 2.8 Determination of Thermal and Structural Properties

The thermal and structural properties of the composite flours were investigated using thermogravimetric analysis (TGA), differential scanning calorimetry (DSC), and Fourier‐transform infrared spectroscopy (FT‐IR), following the methods of Mshayisa et al. (2021) with slight modifications.

#### 2.8.1 Thermo‐Gravimetric Analysis (TGA)

Approximately 4–6 mg of each composite flour sample was weighed into an aluminium pan. A sealed, empty pan was used as a reference. The TGA system (TGA‐7, Perkin Elmer, Singapore) was used to evaluate the thermal stability behavior of the composite flours, where analysis was performed under conditions of 30°C–650°C at a heating rate of 20°C/min and using carrier gas nitrogen, flowing at a rate of 50 mL/min.

#### 2.8.2 Differential Scanning Calorimetry (DSC)

Following a method similar to TGA, approximately 4–6 mg of each composite flour sample was weighed into an aluminium pan. A sealed, empty pan was used as a reference. The DSC profile of the composite flour samples was evaluated using a thermal analyzer (Q2000, Perkin Elmer, Singapore), where analysis was performed under conditions of 30°C–180°C at a heating rate of 20°C/min. The thermal transitions (endothermic and exothermic) were observed, and the onset, peak, and end‐set temperatures, along with the change in enthalpy (∆*H*), were determined.

#### 2.8.3 Fourier‐Transform Infrared Spectroscopy (FT‐IR)

For the structural analysis of the composite flours, an FT‐IR equipped with a universal attenuated total reflectance (UATR) polarization accessory (Spectrum 400, PerkinElmer, Singapore) was used. Prior to the analysis, a background spectrum was obtained. The analysis was conducted by covering the ATR crystal surface with the flour samples and then obtaining spectra by combining 32 scans over 400–4000 cm^−1^ at a resolution of 4 cm^−1^. Between measurements, the UATR crystal was cleaned with acetone to prevent cross‐contamination and ensure accurate spectral acquisition.

### 2.9 Data Analysis

The experiment was replicated, and statistical analysis was conducted using SPSS software version 29 (SPSS Inc., Chicago, IL, USA). All analyzes were conducted in triplicate, unless stated otherwise. Values were expressed as mean ± standard deviation. Multivariate analysis of variance (MANOVA) was utilized to determine mean differences (*p* < 0.05) between treatments, and Duncan's multiple range test was performed to separate the means when differences were found.

## 3 Results and Discussion

### 3.1 Proximate Composition of Wheat‐Mopane Composite Flours

The proximate composition of the composite flours enriched with MWF is presented in Table 1. The incorporation of MWF significantly altered the nutritional profile of wheat flour, with distinct trends observed in crude protein, crude fat, total ash, carbohydrates, and energy contents. The substitution of WF with MWF resulted in a statistically significant (*p* < 0.05) increase in crude protein content across the composite formulations. Protein levels increased from 14.99% in 100% WF to 21.07% at 20% substitution, with native MWF exhibiting the highest protein concentration at 43.39%. These findings underscore the protein‐dense nature of MWF and its potential as a functional ingredient in protein‐enriched food formulations. Although no significant differences (*p* > 0.05) were observed between the WF and MW5%, nor between the 10% and 15% composite flours, all formulations containing 5%–15% MWF differed significantly (*p* < 0.05) from the 20% substitution level, indicating a threshold at which MWF meaningfully enhances protein quantity. These outcomes corroborate previous findings demonstrating that incremental incorporation of edible insect flours elevates protein levels in food matrices (Xie et al. 2024; Zielińska et al. 2021). This study extends beyond earlier work that has predominantly evaluated mopane worm powder as an isolated ingredient (Mashau et al. 2026; Vanqa et al. 2022), by examining its behavior within wheat‐based composite systems. Such an approach is critical, as wheat remains a globally dominant cereal in baked and processed foods, and partial substitution with nutrient‐dense insect flours offers a more realistic pathway toward improving protein intake in human diets. Collectively, the findings affirm the high protein value of yellow mopane worm powder and highlight its potential as an accessible, sustainable protein source, particularly in regions where conventional animal‐derived proteins remain economically or logistically inaccessible (Mashau et al. 2026).

**TABLE 1:** Proximate composition of the composite flours enriched with mopane worm powder.

Flour samples^†^	Crude protein (%)	Crude fat (%)	Total ash (%)	Moisture (%)	Carbohydrates (%)	Energy (kCal/100 g)
WF	14.99 ± 0.26^a^	0.18 ± 0.09^a^	1.05 ± 0.08^a^	8.42 ± 5.75^a^	75.36 ± 6.15^b^	363.04 ± 22.86^a^
MWF	43.39 ± 0.96^d^	5.22 ± 1.12^c^	7.97 ± 2.92^b^	8.49 ± 0.15^a^	34.94 ± 2.58^a^	360.25 ± 16.84^a^
MW5%	15.37 ± 0.56^a^	0.27 ± 0.04^a^	1.41 ± 0.12^a^	9.65 ± 0.17^a^	64.12 ± 16.48^b^	320.38 ± 68.15^a^
MW10%	17.75 ± 0.54^b^	0.76 ± 0.11^a^	1.83 ± 0.17^a^	10.28 ± 2.23^a^	69.88 ± 3.25^b^	357.35 ± 14.64^a^
MW15%	18.92 ± 0.17^b^	1.13 ± 0.56^ab^	2.16 ± 0.14^a^	9.04 ± 0.35^a^	75.67 ± 10.43^b^	388.48 ± 43.22^a^
MW20%	21.07 ± 1.40^c^	1.96 ± 0.21^b^	2.71 ± 0.17^a^	8.55 ± 0.11^a^	67.22 ± 1.07^b^	370.86 ± 3.10^a^

*Note:* Values are mean ± standard deviation (*n = 3*). Mean values within the same column followed by a different superscript indicate significant differences (*p* < 0.05).

^†^ The description of flour samples: WF = 100% wheat flour; MW5% = composite flour with 5% mopane worm powder; MW10% = composite flour with 10% mopane worm powder; MW15% = composite flour with 15% mopane worm powder; MW20% = composite flour with 20% mopane worm powder; MWF = 100% mopane worm powder.

Crude fat content demonstrated a statistically significant (p < 0.05) increase with the substitution of WF by MWF, rising from 0.18% in the control (100% WF) to 1.96% in the 20% MWF.

The highest lipid concentration was recorded in 100% MWF, at 5.22%, as shown in Table 1. These findings are consistent with previous reports on MWF and other edible insects, which are naturally rich in essential fatty acids (Numbi Muya et al. 2022). Nevertheless, the fat content in this study was lower than that of black MWF (13.91%) as reported by Vanqa et al. (2022). While elevated lipid levels may raise concerns about oxidative stability and shelf life, they also enhance the caloric density and functional properties of the composite flours, particularly mouthfeel and flavor (Kwiri et al. 2020).

Ash content, which denotes the total inorganic mineral residue remaining after complete combustion, increased with increasing MWF substitution levels. Statistically, no significant (*p* > 0.05) differences were observed between the WF and composite flours; the MWF had a significantly (*p* < 0.05) higher ash content than the other four samples. The WF sample exhibited an ash content of 1.05%, which increased to 2.71% in the 20% MWF substitution level. The highest value was obtained in the 100% MWF sample, with an ash content of 7.97%. These findings are consistent with the existing literature, which identifies MWF as a nutritionally dense species, particularly rich in minerals such as calcium, iron, and zinc (Nantanga and Amakali 2020). Given that ash content serves as a robust indicator of mineral density (Afify et al. 2017), the observed increases suggest that integrating MW into cereal‐based food matrices could provide a practical means to enhance mineral intake and mitigate micronutrient deficiencies in nutritionally at‐risk populations.

Moisture content varied within a narrow range (8.42%–10.28%) (Table 1), with no significant differences (*p* > 0.05) noted between all the flours. Maintaining moisture below 12% is favorable for flour stability, indicating that shelf‐life would not be adversely affected. According to Codex Alimentarius (Codex Standard 152–1985), the maximum allowable moisture in wheat flour is 15.5%; thus, the composite flours fall much lower than the acceptable Codex limit. The low moisture levels suggest that the flours may be shelf‐stable and can be stored long‐term under appropriate conditions (Aydin et al. 2009; Bodor et al. 2024). Moreover, at low moisture levels, flours are less prone to microbial or mycotoxin contamination and to unpleasant flavors and odors (Bodor et al. 2024).

Carbohydrate content demonstrated no significant differences (*p* > 0.05) between the WF and composite flours (MW5%–MW20%); however, a decrease was observed with the addition of MWF from 75.36% in WF to 67.22% in MW20%. This reduction is attributed to the dilution effect of MWF's high protein and fat content. These results are comparable to findings by de Oliveira et al. (2017), who observed reduced carbohydrate fractions in insect‐cereal composites. Such reductions may be advantageous in lowering glycaemic load, thereby contributing to the development of functional foods targeted at consumers with metabolic disorders. The carbohydrate fraction in edible insects is predominantly attributed to dietary fiber, particularly insoluble chitin derived from the insect exoskeleton (Van Huis 2013).

Energy values of the flours ranged between 320.38 and 388.48 kCal/100 g. When observing the energy values between the controls (WF and MWF) and the four composite flours (MW5%, MW10%, MW15%, MW20%). No significant differences were observed (*p* > 0.05), indicating that the flours may provide comparable energy contributions when incorporated into food products. Vanqa et al. (2024) reported findings similar to this study, in which composite flours substituted with 5%, 10%, 15%, and 20% *M. subhylanus* flour showed energy values ranging from 359.53 to 366.92 kCal/100 g. Given that fat, carbohydrates, and proteins are major sources of dietary energy, the presence of these macronutrients in edible insects suggests their potential to contribute meaningfully to the energy value of food products (Hlongwane et al. 2020).

### 3.2 Mineral Composition of Wheat‐Mopane Composite Flours

**FIGURE 1:**
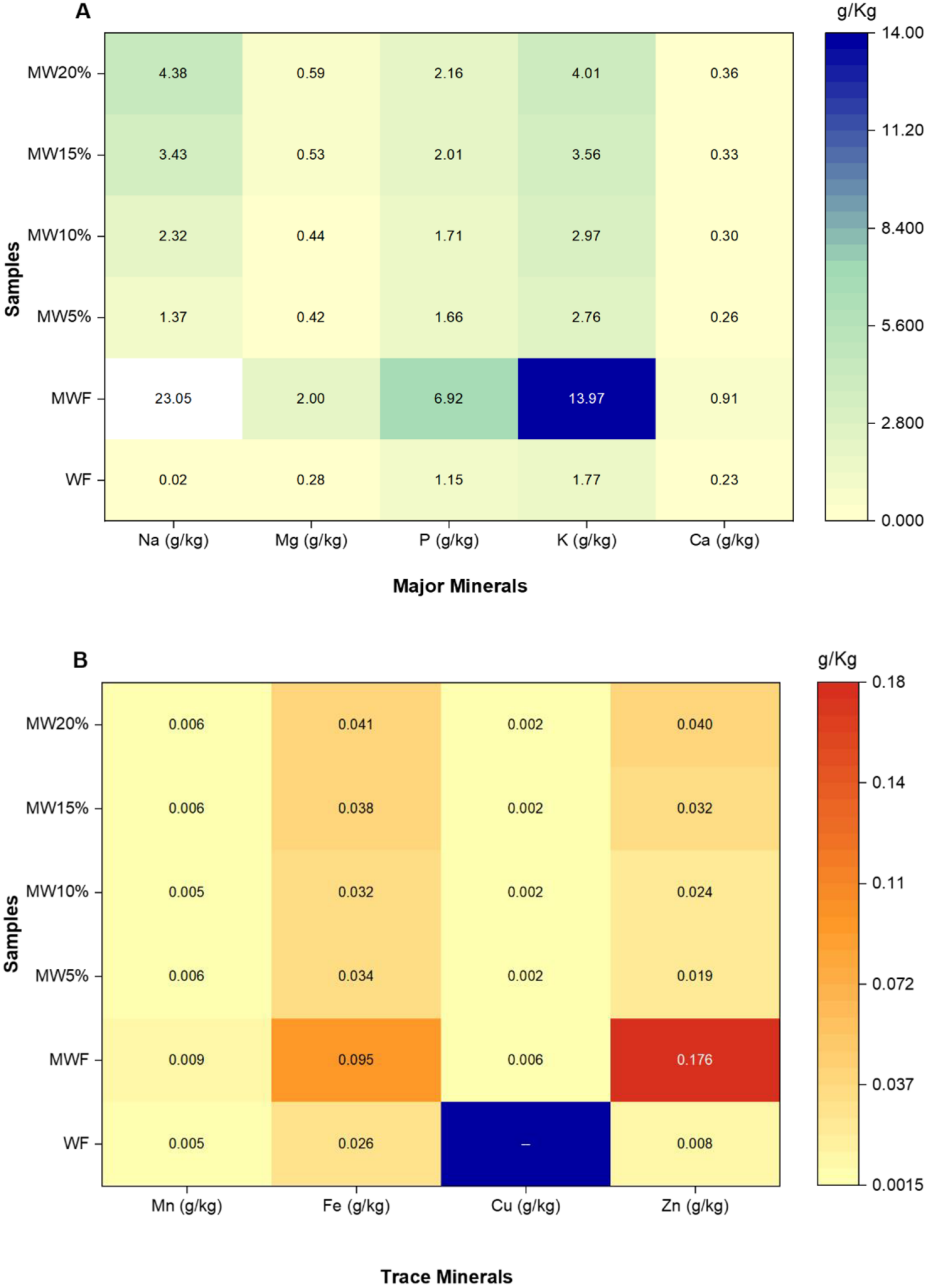
(A) Macro minerals composition of wheat flour (WF), mopane worm powder (MWF), and the composite flours (MW5%–MW20%). (B) Trace minerals composition of wheat flour (WF), mopane worm powder (MWF), and the composite flours (MW5%–MW20%).

Beyond macronutrients, the nutritional value of composite flours is strongly influenced by their mineral content. Mopane worms, traditionally consumed in Southern Africa, are particularly rich in essential minerals, as evidenced by their high total ash content reported by Kwiri et al. (2020) and shown in Table 1. Investigating the mineral composition of wheat‐mopane worm composites provides insights into how insect fortification can address mineral deficiencies while maintaining product functionality. To complement the proximate composition, the macro‐ and trace‐mineral composition of the composite flours was quantified, and their distributions are illustrated in Figure 1A,B as heat maps. As expected, MWF was found to be rich in an array of macro minerals, with Na (23.05 g/kg), Mg (2.00 g/kg), P (6.92 g/kg), K (13.94 g/kg), and Ca (0.91 g/kg) being the dominant minerals. An increase in macro minerals was observed with increasing MWF inclusion in the composite flours (MW5%–MW20%), whereas WF showed significantly lower levels of Na, Mg, P, K, and Ca. These findings agreed with those of Ouma et al. (2022), who reported incremental increases in composite flours enriched with *G. zambesina* at substitution levels of 5%–20%.

On the other hand, the trace minerals reported in the WF, MWF, and composite flours were Mn (0.005 g/kg to 0.009 g/kg), Fe (0.026 g/kg to 0.095 g/kg), Cu (0.002 g/kg to 0.006 g/kg), and Zn (0.008 g/kg to 0.176 g/kg). Wheat flour had the lowest trace mineral values, and the absence of the Cu element, while the composite flours indicated superior trace minerals as the MWF incorporation increased. Based on the findings of this study, incorporating MWF as a functional ingredient would provide a good source of macro‐ and trace minerals that support the body's functions. Although MWF's are nutrient‐dense and demonstrates high mineral concentrations, mineral content does not necessarily equate to mineral bioavailability. There is currently scant literature regarding the bioavailability and absorption of minerals from mopane worm powder. Hence, further studies are required to evaluate mineral bioavailability in MWF‐containing food systems. Conversely, while the insect flour may exceed the recommended daily intake (RDI) for some minerals, such as sodium (approximately 1500 mg),it suggests using it as a food additive to fortify ingredients with much lower mineral content while maintaining the recommended daily mineral intakes at safe levels.

### 3.3 Amino Acid Composition of Composite Flours

As demonstrated in Table 1, the incorporation of MWF into wheat flour resulted in a statistically significant (*p* < 0.05) increase in crude protein content, underscoring the need to examine the nutritional quality of the protein. However, crude protein content alone do not adequately reflect nutritional quality, which is determined by the amino acid composition, particularly the essential amino acids (EAAs) that must be obtained through the diet (Boland et al. 2013; Stamer 2015). Wheat flour is inherently limited in key EAAs such as lysine and threonine (Boye et al. 2010), whereas MWF is known to provide high concentrations of these amino acids (Kouřimská and Adámková 2016). The amino acid composition of wheat‐mopane worm composite flours alongside MWF and WF is exhibited in Table 2. MWF recorded the highest levels of both essential and non‐essential amino acids, with lysine (4.48 ± 0.12 g/100 g) and leucine (4.10 ± 0.06 g/100 g) being the most abundant EAAs. Lysine was identified as the first limiting amino acid in WF, MW5%, and MW10%, consistent with the known cereal limitation (Mshayisa et al. 2022). However, lysine levels in MWF offset this deficiency, and inclusion levels of 15% or more markedly improved amino acid scores (Table 3).

**TABLE 2:** Amino acid profile (g/100 g) of wheat flour, mopane worm powder and composite flours enriched with mopane worm powder.

Essential amino acids									
Flours	Histidine	Isoleucine	Leucine	Phenylalanine	Lysine	Methionine	Valine	Threonine	Sum
WF	0.20 ± 0.00^a^	0.47 ± 0.02^a^	0.86 ± 0.03^a^	0.47 ± 0.04^a^	0.34 ± 0.00^a^	0.19 ± 0.01^a^	0.48 ± 0.01^a^	0.43 ± 0.03^a^	3.45 ± 0.07^a^
MWF	1.45 ± 0.06^d^	2.74 ± 0.05^f^	4.10 ± 0.06^e^	2.94 ± 0.04^f^	4.48 ± 0.12^f^	1.26 ± 0.17^d^	3.08 ± 0.12^e^	2.79 ± 0.07^e^	22.84 ± 0.68^f^
MW5%	0.26 ± 0.00^ab^	0.54 ± 0.00^b^	0.94 ± 0.00^a^	0.58 ± 0.00^b^	0.50 ± 0.00^b^	0.26 ± 0.00^ab^	0.58 ± 0.00^b^	0.55 ± 0.00^b^	4.21 ± 0.00^b^
MW10%	0.36 ± 0.03^bc^	0.68 ± 0.01^c^	1.16 ± 0.02^b^	0.77 ± 0.00^c^	0.63 ± 0.00^c^	0.31 ± 0.01^abc^	0.73 ± 0.01^c^	0.73 ± 0.03^c^	5.37 ± 0.09^c^
MW15%	0.33 ± 0.00^bc^	0.75 ± 0.02^d^	1.36 ± 0.22^c^	0.72 ± 0.03^d^	1.37 ± 0.00^d^	0.33 ± 0.05^bc^	0.78 ± 0.01^c^	0.69 ± 0.00^c^	6.32 ± 0.13^d^
MW20%	0.49 ± 0.00^c^	0.93 ± 0.00^e^	1.55 ± 0.00^d^	1.02 ± 0.00^e^	1.46 ± 0.03^e^	0.44 ± 0.00^c^	0.99 ± 0.00^d^	0.87 ± 0.03^d^	7.65 ± 0.03^e^

Non‐essential amino acids									
Flours	Proline	Arginine	Serine	Glycine	Aspartic acid	Glutamic acid	Alanine	Tyrosine	Sum
WF	1.46 ± 0.01^a^	0.44 ± 0.04^a^	0.67 ± 0.02^a^	0.44 ± 0.04^a^	0.54 ± 0.04^a^	4.67 ± 0.00^c^	0.36 ± 0.01^a^	0.27 ± 0.02^a^	8.86 ± 0.19^a^
MWF	3.20 ± 0.35^b^	3.37 ± 0.06^e^	2.32 ± 0.06^d^	2.57 ± 0.23^d^	4.55 ± 0.35^c^	6.70 ± 0.23^e^	2.86 ± 0.06^f^	3.96 ± 0.01^e^	29.53 ± 0.05^e^
MW5%	1.46 ± 0.00^a^	0.53 ± 0.00^b^	0.72 ± 0.00^ab^	0.52 ± 0.00^ab^	0.74 ± 0.00^a^	4.65 ± 0.00^bc^	0.47 ± 0.00^b^	0.42 ± 0.00^b^	9.51 ± 0.00^b^
MW10%	1.58 ± 0.03^a^	0.66 ± 0.09^c^	0.80 ± 0.10^b^	0.68 ± 0.01^b^	1.24 ± 0.31^b^	4.93 ± 0.00^d^	0.62 ± 0.02^c^	0.55 ± 0.01^c^	11.06 ± 0.38^c^
MW15%	1.53 ± 0.00^a^	0.66 ± 0.03^c^	0.80 ± 0.01^b^	0.71 ± 0.05^b^	1.25 ± 0.40^b^	4.47 ± 0.06^ab^	0.71 ± 0.02^d^	0.56 ± 0.04^c^	10.69 ± 0.43^c^
MW20%	1.72 ± 0.00^a^	0.85 ± 0.02^d^	0.96 ± 0.01^c^	0.92 ± 0.03^c^	1.33 ± 0.01^b^	4.44 ± 0.00^a^	0.84 ± 0.02^e^	0.77 ± 0.00^d^	11.83 ± 0.05^d^

*Note:* Values are mean ± standard deviation (*n = 3*). Mean values within the same column followed by a different superscript indicate significant differences (*p* < 0.05). The description of flour samples: WF = 100% wheat flour; MW5% = composite flour with 5% mopane worm powder; MW10% = composite flour with 10% mopane worm powder; MW15% = composite flour with 15% mopane worm powder; MW20% = composite flour with 20% mopane worm powder; MWF = 100% mopane worm powder.

**TABLE 3:** Amino acid scores of composite flours enriched with mopane worm powder.

Amino acids	FAO/WHO/UNU 2007 (mg/g protein)	WF	MWF	MW5%	MW10%	MW15%	MW20%
Histidine	15	109.6	182.2	126.3	140.3	132.0	167.8
Threonine	23	146.6	230.1	174.3	193.7	180.0	198.7
Lysine	45	62.1^a^	188.6	81.0^a^	86.6^a^	197.3	156.4
Methionine	16	92.5^b^	139.5	118.4	116.0	112.5	141.2
Valine	39	101.2	148.5	108.4	115.8	118.4	130.4
Isoleucine	30	126.1	173.8	131.2	138.2	148.0	159.2
Leucine	59	117.1	132.7	116.1	119.6	124.0	134.9
Phenylalanine + Tyrosine	38	106.0	147.9	111.2	125.4	110.5	137.9
Total EAA	275	861.3	1343.2	967	1035.6	1122.7	1226.7

^a^ First limiting amino acid.

^b^ Second limiting amino acid.

Similarly, threonine was adequately supplied in MWF (2.79 ± 0.07 g/100 g), further complementing cereal proteins. Histidine (1.45 ± 0.06 g/100 g) and methionine (1.26 ± 0.17 g/100 g) were comparatively lower in MWF, yet these limitations are less critical in composite systems. Histidine is particularly pertinent in the growth and development of infants and young children (Mashau et al. 2026). Conversely, cysteine and tryptophan were not detected in the composite flours and control samples, likely due to degradation of these amino acids during the acid hydrolysis used in the analysis. Consequently, future investigations using alternative analytical methods are warranted to accurately quantify these amino acids in MWF and to evaluate their potential as a suitable substitution, particularly given that tryptophan has been reported as a limiting amino acid in cereal proteins (Carcea 2020).

The total essential amino acid (EAA) content of MWF (22.84 g/100 g) was slightly below the FAO/WHO/UNU 2007 recommendations of 27.5 g/100 g (FAO 2013), yet the flour provided excellent levels of lysine, valine, threonine, and histidine, which are often limiting in cereal‐based diets. Although the FAO (2013) report introduced the Digestible Indispensable Amino Acid Score (DIAAS), numerous studies (Miankeba et al. 2022; Perez‐Santaescolastica et al. 2023; Zielińska et al. 2015) post 2013 on edible insect protein quality continue to apply amino acid score (AAS) and protein digestibility‐corrected amino acid score (PDCAAS) approaches based on the FAO/WHO/UNU (2007) reference pattern. Moreover, data on the digestibility of amino acids from edible insect species remains sparse. Hence, the use of the FAO (2007) amino acid scoring pattern in the present study remains an appropriate benchmark to assess the amino acid composition in the composite flours, MWF and WF. What distinguishes the present study from previous work is its focus on the amino acid composition of composite flours rather than individual insect powders. By demonstrating that the inclusion of MWF in wheat flour corrects lysine deficiency and improves the overall amino acid balance, this study moves beyond compositional characterization to application‐driven innovation in staple food systems.

On the non‐essential amino acids, glutamic acid and aspartic acid were predominant in all samples, with MWF showing significantly higher (*p* < 0.05) levels than the composite flours (MW5%–MW20%) and WF. While these amino acids are synthesized by the human body, they are well known for enhancing the umami taste of foods. This suggests that MWF not only contributes to the nutritional improvement of composite flours but may also enhance their sensory properties, thereby supporting consumer acceptability and the development of insect‐fortified cereal products.

### 3.4 Physicochemical Properties

#### 3.4.1 Color Characteristics of the Composite Flours

The color attributes of MWF and wheat‐mopane composite flours were evaluated using the CIE Lab* system (Table 4). Lightness (L*) declined significantly (*p* < 0.05) with higher mopane worm inclusion, from 89.53 in WF to 60.13 in MWF. The progressive darkening indicates the dominance of the insect powder's intrinsic pigments and protein‐lipid complexes, which reduce reflectance and yield a visually darker composite flour. Consistent with this finding, Andrzejczyk et al. (2024) reported that substituting yoghurt with 1.5%–5% yellow mealworm powder significantly reduced product brightness, attributing the effect to the pigment‐rich and proteinaceous nature of the insect flour. The redness (+a*) and yellowness (+b*) values of the flours increased significantly (*p* < 0.05) with higher MWF substitution, while no samples exhibited negative a* or b* values, indicating the absence of green or blue hues.

**TABLE 4:** Physicochemical properties of the composite flours enriched with mopane worm powder.

Physicochemical component	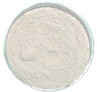	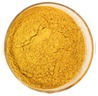	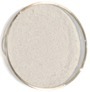	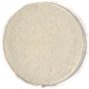	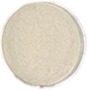	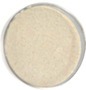
	WF (Control)	MWF	MW5%	MW10%	MW15%	MW20%
L*	89.53 ± 0.09^f^	60.13 ± 0.24^a^	84.99 ± 0.18^e^	83.35 ± 0.08^d^	81.60 ± 0.23^c^	79.98 ± 0.11^b^
a*	0.43 ± 0.09^a^	5.73 ± 0.11^f^	0.88 ± 0.09^b^	1.13 ± 0.03^c^	1.60 ± 0.08^d^	1.87 ± 0.03^e^
b*	7.72 ± 0.06^a^	27.10 ± 0.34^f^	11.16 ± 0.19^b^	12.19 ± 0.25^c^	13.70 ± 0.41^d^	14.66 ± 0.12^e^
∆*E*	Control	35.61 ± 0.34^f^	5.72 ± 0.02^b^	7.66 ± 0.20^c^	10.01 ± 0.38^d^	11.90 ± 0.16^e^
Bulk Density (g/mL)	0.76 ± 0.06^c^	0.57 ± 0.04^a^	0.66 ± 0.01^b^	0.66 ± 0.02^b^	0.67 ± 0.02^b^	0.70 ± 0.01^b^
A_w_	0.59 ± 0.01^b^	0.56 ± 0.00^a^	0.63 ± 0.02^d^	0.62 ± 0.01^cd^	0.62 ± 0.00^cd^	0.60 ± 0.01^bc^
pH	5.82 ± 0.06^ab^	6.12 ± 0.02^d^	5.77 ± 0.03^a^	5.85 ± 0.04^b^	5.92 ± 0.04^c^	5.97 ± 0.02^c^

*Note:* Values are mean ± standard deviation (*n = 3*). Mean values within the same row followed by a different superscript indicate significant differences (*p* < 0.05). The description of flour samples: WF = 100% wheat flour; MW5% = composite flour with 5% mopane worm powder; MW10% = composite flour with 10% mopane worm powder; MW15% = composite flour with 15% mopane worm powder; MW20% = composite flour with 20% mopane worm powder; MWF = 100% mopane worm powder.

The MWF recorded the highest a* and b* values (5.73 and 27.10, respectively), reflecting its intrinsic reddish‐yellow pigmentation. Accordingly, the composite flours showed increased color saturation within the red‐yellow spectrum relative to the control, indicating that contribution of MWF pigments and associated macromolecules to the overall visual quality of the flour.

The observed increase in both parameters confirms the pigment‐rich nature of MWF, whose carotenoids, Maillard‐derived compounds, and surface lipoproteins contribute to enhanced chromatic intensity. In this study, ΔE values ranged from 5.72 (MW5%) to 35.61 (MWF), indicating highly visible and statistically significant (*p* < 0.05) differences across formulations. These pronounced shifts in color intensity reflect the strong pigmentation of MWF and its dominant influence on the composite flours' optical properties. This study is among the first to characterize the extent of color divergence in mopane worm‐wheat composites, providing a new understanding of how entomological ingredients modulate the visual quality of cereal systems. From an industrial perspective, such data are essential for product standardization, as color uniformity affects consumer trust and perceived freshness. Furthermore, the distinct colouration of mopane‐based flours may offer functional opportunities for product differentiation, such as in rustic, wholegrain‐style, or high‐protein baked goods, where darker tones are associated with naturalness and nutritional density.

#### 3.4.2 Bulk Density

The bulk density of the flours was determined, and the results are presented in Table 4. Bulk density, a concept used to describe the weight of powder per unit volume, is influenced by factors such as particle size and initial moisture content, which affect the particle arrangement of the product (Chandra et al. 2015). The bulk density of the flours ranged from 0.57 g/mL to 0.76 g/mL, with the MWF having the lower value and the WF the higher. Furthermore, composite flours had lower values than WF. The reduced bulk density in composite flours is associated with the addition of MWF, which may reduce starch content, thereby limiting the formation of a starch‐based matrix that contributes to flour density (Wójtowicz et al. 2023). Furthermore, the mechanism extends to protein interactions, where MWF may have disrupted the glutenin and gliadin in wheat flour by weakening their covalent and hydrogen‐bond networks (Roncolini et al. 2019; Tanga et al. 2025). Vanqa et al. (2022) reported a bulk density of 0.65 g/mL black MWF, and these differences may be attributed to the various diets and seasons the mopane worms may have been exposed to. Bulk density plays a pivotal role in determining packaging requirements, material handling, and the application of a product in the manufacturing of certain foods (Mshayisa et al. 2022). Therefore, the lower bulk density as determined in MWF and composite flours suggests ease in packability, storage, and transportation.

#### 3.4.3 Water Activity (Aw) and pH

This study also aimed to assess the water activity and pH of the composite flours to determine the effect of incorporating mopane worm powder on their physicochemical stability and potential shelf life (Table 4). Water activity (A_w_) is crucial in determining shelf stability, as it predicts the growth of bacteria, yeasts, and molds by indicating the amount of water available in food for these organisms to grow and potentially (Sandulachi and GhTatarov 2012). On the other hand, pH is another important factor to consider for shelf stability and food safety, as controlling food acidity can help limit potential microbial growth.

As seen in Table 4, the pH values of the composite flours ranged from 5.77 to 5.97. Among the composite flour samples, MW15 and MW20 exhibited significantly higher pH values (*p* < 0.05) compared to MW5 and MW10. Conversely, the pH of WF did not differ significantly (*p* > 0.05) from those of MW5 and MW10, whereas MWF exhibited a significantly higher pH than all the flour samples evaluated. A study conducted by Vanqa et al. (2024) reported similar pH values (< 0.60) in composite flours enriched with *M. subhylanus* at 5%–20% incorporation. Conversely, a decrease in a_w_ from 0.63 (MW5%) to 0.60 (MW20%) was observed in the composite flours, as the MWF incorporation increased. These findings suggest that under proper storage, fortifying wheat flour with MWF at 5% to 20% would have reduced susceptibility to bacterial growth and increased shelf stability, contributing to the potential hurdle effect of the low pH and A_w_ (< 0.60). Moreover, these composite flours propose a nutritious, food‐safe and low‐cost alternative, especially in low‐income households where wheat‐based flours are a staple ingredient.

### 3.5 Techno‐Functional Properties

#### 3.5.1 Water and Oil‐Binding Capacity

Water‐binding capacity (WBC) and oil‐binding capacity (OBC) are critical functional properties that influence the texture, mouthfeel, stability, and sensory quality of food systems. WBC reflects the ability of proteins to retain water through mechanisms such as dissolution and gel formation, whereas OBC describes the capacity to absorb and retain lipids, which contributes significantly to flavor retention and palatability (Vanqa et al. 2022; Zhang et al. 2023). These functional attributes are strongly influenced by intrinsic protein characteristics, including amino acid composition, protein conformation, surface hydrophobicity, and polarity (Akpossan et al. 2015; Mshayisa et al. 2022). The present study evaluated the WBC and OBC of wheat flour (WF), yellow mopane worm powder (MWF), and their composite blends, and the findings are presented in Table 5.

**TABLE 5:** Techno‐functional properties of the composite flours enriched with mopane worm powder.

Flour samples^†^ ^s^	Water‐binding capacity (g/g)	Oil‐binding capacity (g/g)	Emulsion capacity (%)	Emulsion stability (%)	Foaming capacity (%)	Foaming stability (%)
WF	1.76 ± 0.08^a^	1.90 ± 0.03^a^	61.54 ± 0.00^b^	58.33 ± 7.22^d^	33.33 ± 17.56^b^	88.62 ± 1.54^bc^
MWF	2.89 ± 0.15^b^	3.79 ± 0.40^b^	47.01 ± 14.12^a^	26.39 ± 20.55^a^	16.67 ± 2.89^a^	86.62 ± 5.42^c^
MW5%	1.73 ± 0.09^a^	1.93 ± 0.06^a^	61.26 ± 0.48^b^	52.94 ± 5.09^cd^	31.67 ± 5.77^ab^	79.95 ± 7.21^a^
MW10%	1.85 ± 0.12^a^	1.87 ± 0.09^a^	60.50 ± 1.15^b^	46.94 ± 3.13^bcd^	26.67 ± 2.89^ab^	82.87 ± 2.51^ab^
MW15%	1.70 ± 0.02^a^	3.92 ± 0.06^b^	58.97 ± 4.44^b^	35.12 ± 9.16^abc^	21.67 ± 5.77^ab^	86.43 ± 5.81^abc^
MW20%	1.78 ± 0.05^a^	3.83 ± 0.20^b^	57.23 ± 3.34^ab^	29.87 ± 7.07^ab^	21.67 ± 5.77^ab^	90.43 ± 2.13^bc^

*Note:* Values are mean ± standard deviation (*n = 3*). Mean values within the same row followed by a different superscript indicate significant differences (*p* < 0.05).

^†^ The description of flour samples: WF = 100% wheat flour; MW5% = composite flour with 5% mopane worm powder; MW10% = composite flour with 10% mopane worm powder; MW15% = composite flour with 15% mopane worm powder; MW20% = composite flour with 20% mopane worm powder; MWF = 100% mopane worm powder.

MWF exhibited a significantly higher (*p* < 0.05) WBC of 2.89 g/g, compared to WF and the composite flours. In contrast, WF and composite flours showed no significant differences (*p* > 0.05) in WBC, with values ranging from 1.70 to 1.85 g/g. It can be deduced that the enhanced WBC of MWF compared to the wheat flour and composite blends is attributed to its porous chitin network and elevated levels of hydrophilic amino acids, which facilitate entrapment and retention of water molecules. The amino acid results in Table 2 further highlight the high values of hydrophilic amino acids in MWF. Comparable WBC values have been reported for defatted *Hermetia illucens* flour (3.0 g/g), indicating that high‐protein insect powders often display strong hydration properties (Mshayisa et al. 2022). The significantly (p < 0.05) high WBC suggests that MWF is well‐suited for moisture‐rich food applications, such as doughs, porridges, and rehydratable mixes. In a study conducted on rheological and textural properties of Chapatti (Roti) enriched with 5%–15% of cricket flour, Khatun et al. (2021) demonstrated that the water absorption, dough storage modulus, dough extensibility, and gluten strength increased with the increase in insect flour.

In terms of OBC, MWF and the MW15% and MW20% composite flours demonstrated significantly higher (*p* < 0.05) OBC values (3.79–3.92 g/g) than WF, MW5%, and MW10% (1.87–1.93 g/g). These results exceed reported OBC values for *Tenebrio molitor* (1.71 g/g) and for several plant‐based proteins, such as soy, lentils, and chickpeas (0.84–1.10 g/g) (Zielińska et al. 2018). The improved OBC at substitution levels ≥ 15% is attributed to the higher concentration of hydrophobic amino acids in MWF, which enhances protein‐lipid interactions and promotes oil retention (Ohizua et al. 2017; Zielińska et al. 2021). This suggests that MWF incorporation could be advantageous in baked products, emulsified foods, and meat analogues, where oil retention for flavor and mouthfeel is critical (Akpossan et al. 2015).

Collectively, the significant improvements in both WBC and OBC highlight the dual functional benefits of MWF in composite cereal systems. To the best of our knowledge, limited studies in South Africa have concurrently reported statistically significant enhancements in both hydration and lipid‐binding properties in mopane‐enriched wheat flours, underscoring the novelty of these findings. These results reinforce the potential of MWF as a functional, sustainable, and commercially viable ingredient for food innovation.

#### 3.5.2 Emulsion Capacity and Stability

Emulsifying capacity (EC) and emulsion stability (ES) are key techno‐functional properties that describe a molecule's ability to facilitate the dispersion of immiscible phases, such as oil and water, and to maintain the structural integrity of the resulting emulsion (López‐Marcos et al. 2015). Protein hydrophobicity plays a central role in emulsion performance, as increased surface hydrophobicity enhances protein‐lipid interactions and promotes the formation of stable emulsions (Zhang et al. 2023). These functional characteristics are essential in various food systems, including dressings, mayonnaise, meat analogues, and batter formulations (Mshayisa and Van Wyk 2021).

The EC and ES of the flours were examined, and the results are illustrated in Table 5. EC decreased progressively as MWF incorporation increased. WF and the composite flours (MW5%–MW20%) exhibited similar EC values, ranging from 58.97% to 61.54%, whereas pure MWF showed the lowest EC (47.00%). This reduction is attributed to the lower proportion of hydrophobic amino acids in MWF, as indicated in Table 2, which limits the protein's capacity to interact effectively with lipids. Comparable trends were reported by Akpossan et al. (2015), who observed reduced EC in flours with higher insect protein content due to diminished surface hydrophobicity.

ES also decreased with increasing MWF substitution, ranging from 52.94% to 29.87% in the composite flours. WF, MW5% and MW20% displayed comparatively superior ES, whereas MWF alone exhibited an ES of 26.37%, consistent with findings in house cricket (21.86%) reported by Ndiritu et al. (2017). These findings indicate that the insect protein demonstrated a weaker oil‐binding interfacial strength, which resulted in the imminent destabilization of the emulsions as MWF increased. The reduction in ES is likely due to partial protein denaturation and weakened gluten networks as WF content declines (Lucas‐González et al. 2019). Notably, Bresciani et al. (2022) also reported decreased emulsifying activity in composite flours with cricket powder; however, they noted that bread enriched with cricket powder up to 10% depicted a similar bread volume and crumb hardness to the control samples without the insect powder. Nevertheless, while MWF provides notable nutritional benefits, its reduced emulsifying performance suggests that partial incorporation with WF may be necessary to optimize its functionality in emulsion‐based food applications.

#### 3.5.3 Foaming Capacity and Stability

Foaming properties of food systems are largely dependent on the proteins present and other components, such as carbohydrates. Various factors aid in characterizing a protein as a foaming agent, including the rate of the protein's migration to the air‐water interface, its capacity to unfold and readapt at the interface, and the physical characteristics of the viscoelastic film produced (Lucas‐González et al. 2019; Sreerama et al. 2012).

The foaming capacity (FC) and foaming stability (FS) of the flours were evaluated, and findings are depicted in Table 5. Significant differences (*p* < 0.05) were observed in FC between MWF (16.67%) and WF (33.33%), while composite flours depicted similarities in FC with both WF and MWF; therefore, indicating that the interaction of these two protein sources may still enhance FC properties. The reduced FC in MWF could be attributed to its limited solubility and tightly aggregated protein structures, which hinder surface denaturation and impede interaction at the air‐water interface (Akpossan et al. 2015). Although MWF exhibited higher FC than previously reported values for the same species (5.81%) (Vanqa et al. 2022), it is still considerably lower compared to the FC of *H. illucens* (40%–78.43%) reported by Mshayisa et al. (2022). Jung et al. (2005) suggest that FC may be improved through heat‐induced denaturation, which would increase the adsorption at the air‐water interface.

Despite the low FC values, all flours exhibited excellent foaming stability, with results ranging from 79.95% (MW5%) to 90.43% (MW20%). This stability is likely due to the rigid protein‐chitin network in MWF, which supports foam structure once formed. Although MW5% showed reduced FC compared to WF, this may reflect weakened protein–protein interactions within the wheat matrix. Nonetheless, these interactions are significantly influenced by the type of insect flour. Zielińska et al. (2023) demonstrated that cricket flour has a better foaming property than worm flours. Similar findings were reported by Mishyna et al. (2021), who noted that cricket powder exhibited a stable foam (86%) compared to yellow peas (49%) and faba beans (62%), thereby attesting to the resilience of insect protein structure, which keeps the air‐water interface intact.

The present study highlights that while MWF may require formulation support to enhance the aeration in food, the robust protein structure provides foam longevity, which may be useful, especially in heat‐processed or whipped food systems.

#### 3.5.4 Pasting Properties of the Composite Flours

Pasting behavior is a fundamental parameter for predicting gelatinization dynamics and understanding the physicochemical interactions that govern the functional performance of flour‐based systems. These properties, which develop during the heating of starch‐water mixtures, influence sensory attributes, processing behavior, and overall applicability in industrial formulations (Vanqa et al. 2024). The pasting properties of the insect‐enriched composite flours are depicted in Table 6, where the peak viscosity (PV), trough viscosity (TV), breakdown viscosity (BV), final viscosity (FV), and setback viscosity (SV) are reported.

**TABLE 6:** Pasting properties of the composite flours enriched with mopane worm powder.

Flour samples^†^	Peak viscosity (cP)	Trough viscosity (cP)	Breakdown viscosity (cP)	Final viscosity (cP)	Setback viscosity (cP)	Peak time (min)	Pasting temperature (°C)
WF	1493.33 ± 246.52^c^	894.67 ± 182.51^b^	598.67 ± 64.17^e^	1927.67 ± 234.11^d^	1033.00 ± 71.04^e^	9.11 ± 0.14^b^	60.33 ± 2.60^a^
MWF	21.00 ± 37.24^a^	−1.33 ± 2.08^a^	22.33 ± 35.22^a^	1.00 ± 1.73^a^	2.33 ± 0.58^a^	3.36 ± 0.50^a^	—
MW5%	1530.67 ± 435.11^c^	923.00 ± 112.19^b^	607.67 ± 122.94^e^	1886.67 ± 140.18^d^	936.67 ± 28.04^de^	9.07 ± 0.18^b^	87.07 ± 0.71^d^
MW10%	1238.67 ± 254.38^bc^	812.33 ± 119.68^b^	426.33 ± 47.08^d^	1579.33 ± 181.27^c^	767.00 ± 113.27^c^	8.89 ± 0.11^b^	75.45 ± 21.18^c^
MW15%	1259.33 ± 26.84^bc^	692.33 ± 124.01^c^	567.00 ± 13.45^c^	1505.67 ± 50.12^c^	813.33 ± 41.36^cd^	9.02 ± 0.15^b^	64.43 ± 0.28^b^
MW20%	945.67 ± 145.96^b^	666.67 ± 130.27^c^	279.00 ± 26.66^b^	909.33 ± 61.16^b^	242.67 ± 153.58^b^	8.67 ± 0.07^b^	62.45 ± 1.89^a^

*Note:* Values are mean ± standard deviation (*n = 3*). Mean values within the same column followed by a different superscript indicate significant differences (*p* < 0.05).

^†^ The description of flour samples: WF = 100% wheat flour; MW5% = composite flour with 5% mopane worm powder; MW10% = composite flour with 10% mopane worm powder; MW15% = composite flour with 15% mopane worm powder; MW20% = composite flour with 20% mopane worm powder; MWF = 100% mopane worm powder.

A significant decline (*p* < 0.05) in PV, BV, and SV was observed with increasing MWF substitution, while TV showed no significant differences among the composite blends (*p* > 0.05).

PV values ranged from 945.67 to 1530.67 cP, with MW5% significantly higher (*p* < 0.05) than MW20%. The reduction in PV at higher MWF levels is attributed to a decreased starch fraction and to competitive interactions between amylose, phospholipids, and proteins, which restrict starch swelling and enhance hydrogen bonding (Gull et al. 2018). These findings align with Carpentieri et al. (2024), who similarly reported PV reductions when insect flours replaced starch‐rich cereal fractions. Emulating similar behavior to the PV, the trough viscosity (TV) was noted to diminish in the composite flours as MWF increased; however, all composite flours did not possess statistically different (*p* > 0.05) trough viscosities, as they ranged from 666.67 cP to 923.00 cP. The MW5% composite blend exhibited the highest TV, attributable to the high starch content.

Meanwhile, as the MWF concentration increased in the 10%, 15% and 20% composite flours the increasing protein content competed with the starch, thus reducing the TV in the composite flours. The decreased BV, particularly in MW20% (279 cP) compared to MW5% (607.67 cP), suggests enhanced resistance to breakdown under shear and thermal stress, likely due to increased protein presence surrounding starch granules, reinforcing structural stability (Villanueva et al. 2018). Notably, a similar trend was observed for the final (FV) and setback viscosities (SV). Similarly, significant declines (*p* < 0.05) were also observed in the composite blends as the MWF incorporation increased for both FV and SV. FV decreased from 1886.67 cP (MW5%) to 909.33 cP (MW20%), and SV from 936.67 cP to 242.67 cP. These reductions reflect a lower starch content and impaired retrogradation capacity during cooling, as noted by Xie et al. (2024) and Vanqa et al. (2024).

Pasting temperature (PT) decreased as MWF increased, ranging from 62.45°C to 87.07°C, likely due to reduced starch and elevated protein and fiber fractions competing for water (Khatun et al. 2021). Peak time, however, did not differ significantly (*p* > 0.05) among the composite flours, whereas MWF alone showed a reduced peak time, likely due to the lack of starch granules.

As insect powder generally possesses distinctively low starch content and is rich in protein, there is a diminished presence of the amylose molecules that aid in forming a stable gel network during heating due to the granule swelling and amylose lipid‐complex formation (Wani et al. 2013). Similarly, Xie et al. (2022) reported reduced pasting properties in dough and bread samples enriched with 5%–20% *T. molitor* (mealworm) powder compared to samples without insect powder, attributing this to starch dilution. Hence, these findings indicate that blending MWF with wheat flour at 5% and 10% provides PV, TV, and SV comparable to 100% wheat flour, making these composite blends suitable in dough applications. Conversely, the blends with 15% and 20% MWF showed reduced susceptibility to breakdown and greater resistance to heating and shear forces, suggesting that the high protein content may reinforce the structure of the gelatinized starch molecules.

### 3.6 Thermal and Structural Properties of the Composite Flours

#### 3.6.1 Thermo‐Gravimetric Analysis (TGA)

**FIGURE 2:**
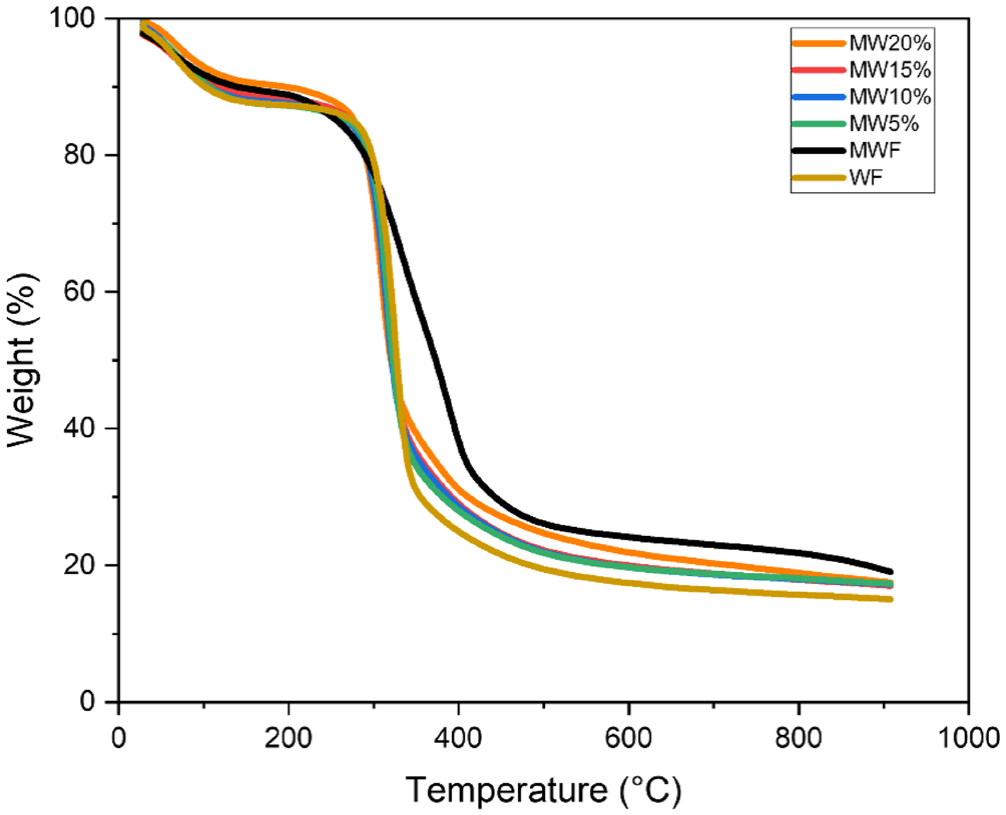
Thermogravimetric (TGA) curves of wheat flour (WF), Mopane worm powder (MWF), and composite flours (MW5%–MW20%).

Thermal stability is a critical property in the functional evaluation of novel food ingredients, particularly those intended for use in thermally processed products, such as baked goods or extruded snacks. In this study, thermogravimetric analysis (TGA) was employed to evaluate the thermal degradation behavior of WF, MWF, and their composite blends. As shown in Figure 2, all samples exhibited a characteristic multi‐phase weight‐loss pattern across the temperature range of 25°C–900°C, consistent with the decomposition of moisture and the major biopolymers, including starch, proteins, lipids, and structural carbohydrates.

The initial weight loss observed below 150°C is attributed to the evaporation of free and loosely bound water. This phase was more pronounced in the composite flours, particularly those with higher MWF content, likely due to the hygroscopic nature of insect‐derived proteins and chitin. Similar observations were made by Mshayisa et al. (2022), who reported that edible insect matrices, especially those containing polar amino acid residues and glycosylated structures, tend to retain higher moisture compared to cereal‐based controls.

The primary decomposition event occurred between approximately 250°C and 400°C, corresponding to the breakdown of the organic matrix. For wheat flour, this steep mass loss is primarily attributed to starch gelatinization and degradation, along with some protein denaturation. In contrast, MWF and composite samples exhibited more gradual and thermally delayed decomposition. This shift suggests greater thermal resistance in the presence of MWF. The protein‐rich, chitinous nature of the insect flour likely contributes to its enhanced thermal stability. Chitin, a linear polysaccharide found in insect exoskeletons, degrades at higher temperatures (> 350°C) due to its crystalline structure and strong hydrogen bonding (Yi et al. 2013). Moreover, insect proteins often possess compact tertiary structures and are rich in disulfide bridges, which confer additional heat resistance (Duda et al. 2019).

At temperatures above 500°C, the curves plateaued, indicating the formation of carbonaceous char and residual ash. Notably, composite samples retained a higher final mass than wheat flour, reflecting the mineral‐rich composition of insect flours. Rumpold and Schlüter (2013) reported that edible insects contain elevated levels of calcium, phosphorus, potassium, and magnesium, which remain as thermally stable inorganic residue upon combustion. This finding was consistent across all mopane‐containing blends, with MW20% exhibiting the highest residual weight among the composite flours at ~19%, compared to ~14% in pure wheat flour. MWF demonstrated the highest residual weight across all samples.

The observed thermal trends suggest that increasing MWF incorporation improves the composite flour system's heat tolerance. This is particularly relevant for industrial food processes that require thermal inputs above 200°C. Enhanced thermal resistance could positively influence the stability of nutritional and functional compounds during processing, improve product shelf life, and reduce the formation of undesirable thermal degradation by‐products. From a product development perspective, the inclusion of insect flours, such as MWF, not only improves the protein content and mineral composition but also enhances the thermal robustness of cereal‐based formulations (Phat et al. 2016).

This synergistic effect has been documented in other insect‐cereal systems. For instance, Duda et al. (2019) demonstrated that incorporating cricket and mealworm powders into wheat‐based matrices led to improved baking performance and enhanced structural stability under thermal stress. TGA analysis revealed that integrating MWF into WF significantly alters the thermal degradation profile of the resulting composite blends. The delayed onset of thermal decomposition and higher residual mass indicate improved thermal stability, which can be attributed to the presence of thermally resistant insect macromolecules, such as chitin, and structurally stable proteins. These findings support the feasibility of MWF as a functional ingredient in thermally processed food systems, contributing to the growing body of evidence that highlights the techno‐functional advantages of edible insects in food innovation.

#### 3.6.2 Differential Scanning Calorimetric (DSC) Analysis

Understanding the factors that induce structural alterations in proteins is essential, as such changes can significantly influence a protein's functional properties. Hence, DSC analysis can be used to investigate heat changes and to provide qualitative and quantitative information on a sample's thermal properties (Mshayisa et al. 2021; Queiroz et al. 2021). Changes in the thermodynamic properties of the flours were investigated, and the results are depicted in Table 7. Endothermic and exothermic characteristics were noted in the WF and composite flours; meanwhile, the MWF only exhibited endothermic properties. Heat‐induced protein unfolding and thermal stability were characterized by the onset temperature (T_on_) and the peak/denaturation temperature (T_p_).

**TABLE 7:** Thermal properties of wheat flour (WF), Mopane worm powder (MWF), and composite flours (MW5%–MW20%).

Flour sample	T_on_ (°C)	T_p_ (°C)	T_end_ (°C)	∆*H* (J/g)
Endothermic peaks				
WF	20.67	91.68	146.51	378.18
MWF	18.05	69.96	111.99	396.08
MW5%	20.95	81.04	135.84	372.34
MW10%	22.23	72.2	127.74	287.36
MW15%	20.38	79.46	135.23	338.09
MW20%	20.71	80.58	136.72	339.51
Exothermic peaks				
WF	294.72	310.44	326.46	−137.08
MWF	No reaction	No reaction	No reaction	No reaction
MW5%	289.5	305.19	320.88	−152.73
MW10%	286.95	303.54	319.31	−99.47
MW15%	284.41	299.38	312.65	−135.57
MW20%	281.04	297.37	311.13	−119.05

The present study indicated that the T_p_ ranged from 69.96°C to 91.68°C, with the MWF exhibiting the lowest denaturation temperature and the WF exhibiting the highest. Denaturation involves the disruption of intramolecular bonds and this is an endothermic process. Thus, these findings suggest that the compact MWF protein transforms from an ordered to an unfolded state at significantly lower temperature compared to the composite flours and WF.

Changes in enthalpy (∆*H*) are used to elucidate the energy requirements to disrupt hydrogen bonds and electrostatic interactions, which may subsequently lead to the unfolding of coiled polypeptides (Li et al. 2024) in endothermic reactions. As outlined in Table 7, the change in enthalpy (∆*H*) for the flours depicted endothermic peaks, which ranged between 287.35 J/g and 396.08 J/g, and the highest positive ∆*H* was observed in MWF.

This observation reveals that more energy was required to break the polypeptides in the MWF protein; furthermore, it also demonstrates a strong protein structure and remarkable thermal stability. The TGA analysis, which showed the MWF sample with the highest weight percentage after heating above 500°C, also supported this finding. Moreover, as the temperature increased, exothermic peaks were observed for WF and composite flours, with MW5% exhibiting the largest negative ∆*H*; however, no exothermic reactions were observed in MWF. The addition of MWF offers greater thermal stability and is a suitable functional ingredient in heat‐treated food products. Furthermore, the dual functionality of the composite flours makes them suitable for food systems that undergo endothermic reactions, such as baking and for those that undergo exothermic reactions, such as Maillard reactions.

#### 3.6.3 Fourier‐Transform Infrared Spectroscopy (FT‐IR) Analysis and Functional Groups

**FIGURE 3:**
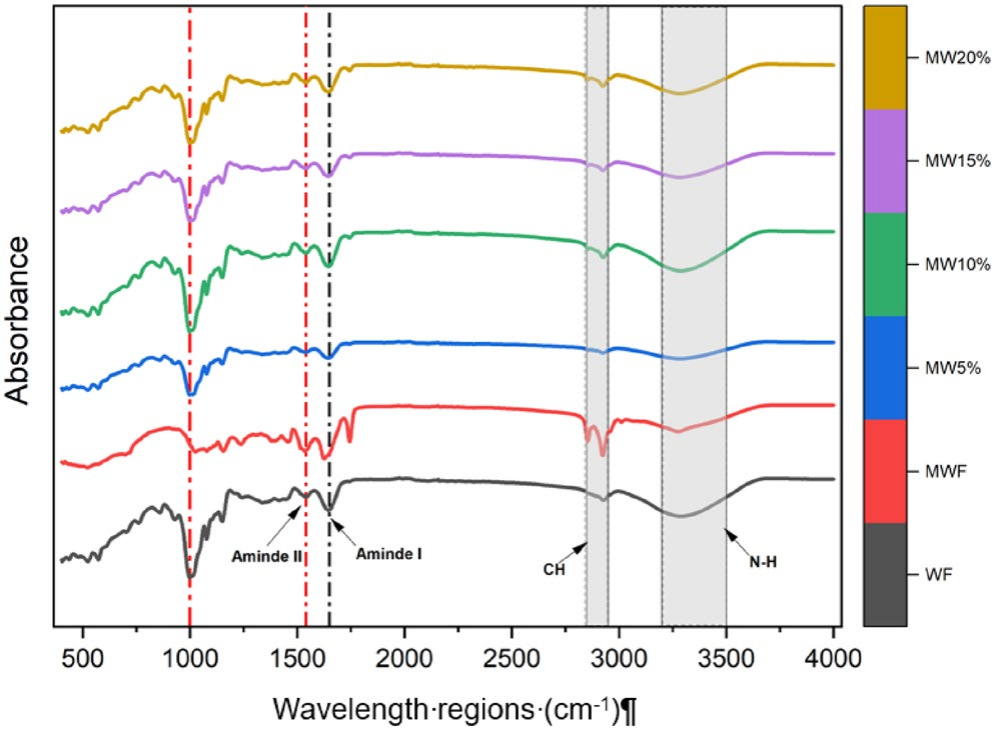
Fourier‐transform infrared spectra of wheat flour (WF), Mopane worm powder (MWF), and composite flours (MW5%–MW20%).

Fourier‐transform infrared spectroscopy (FT‐IR) is a rapid, non‐destructive analytical technique extensively used in food science to elucidate the structural and compositional characteristics of biopolymeric materials. In the present study, FT‐IR analysis was employed to evaluate the molecular interactions and functional group dynamics of MWF, WF, and their respective composite blends (MW5%–MW20%). The FT‐IR spectra (Figure 3) revealed several distinctive absorption bands corresponding to key biochemical constituents. Table 8 further expounds on the structural changes that are expected to occur in each wavelength (cm^−1^) region as a result of the presence of key biochemical constituents. All samples displayed a broad peak between 3200 and 3400 cm^−1^, indicative of O—H and N—H stretching vibrations associated with hydroxyl groups from polysaccharides and proteins. Notably, the intensity of this band was elevated in MWF and composite samples, reflecting greater moisture retention capacity and the presence of protein‐associated amino groups.

**TABLE 8:** Structural changes associated with the wavelength regions obtained on the wheat flour (WF), Mopane worm powder (MWF), and composite flours (MW5%–MW20%) analyzed using FT‐IR spectroscopy.

Region (cm^−1^)	Assignment	Observed in MWF‐rich samples	Interpretation
3200–3500	O—H and N—H stretching (hydroxyl and amide groups)	Broader and more intense	Indicates higher protein and lipid content in Mopane worm powder; hydrogen bonding increases
2850–2950	C—H stretching (aliphatic chains)	More defined peaks in MWF	Associated with lipid chains, confirming higher fat content in MWF
1630–1650	Amide I (C=O stretching of proteins)	Stronger in MWF‐rich blends	Reflects greater protein concentration and changes in secondary structure
1530–1550	Amide II (N—H bending, C—N stretching)	Present mainly in MWF samples	Further supports protein enrichment with Mopane worm powder
1400–1450	CH_3_/CH_2_ bending, protein side chains	Slightly stronger in MWF blends	Related to protein and fatty acid chains
1000–1200	C—O—C and C—O stretching (carbs, polysaccharides)	Minor variation seen	Indicates differences in carbohydrate types between WF and MWF
900–1000	CH deformation (lipids, polysaccharides)	More intense in MWF‐rich samples	Linked to chitin or lipid‐associated peaks in insects

Characteristic amide I (~1650 cm^−1^) and amide II (~1540 cm^−1^) bands were strongly expressed in MWF, signifying prominent C=O stretching and N—H bending vibrations from peptide bonds. In contrast, WF exhibited relatively weak absorption in this region, reflecting its lower protein content. An increase in band intensity was observed in the composite flours as MWF incorporation increased, confirming successful protein fortification and molecular interactions between the two matrices.

Additional peaks in the region of 2800–2950 cm^−1^ were attributed to C—H stretching of aliphatic chains, commonly associated with lipid components. These peaks were more prominent in MWF and the composite flours, consistent with the known lipid‐rich nature of edible insects. Furthermore, intense absorptions between 1000 and 1150 cm^−1^, corresponding to C—O and C—O—C stretching vibrations from carbohydrates and chitin, were observed across all samples, though with broader bands in the insect‐containing flours. This may be linked to the presence of insect‐derived polysaccharides, such as chitin, and their interactions with wheat starch. The inclusion of MWF led to noticeable spectral shifts and increased band intensities in protein‐ and lipid‐associated regions.

These findings agree with previous studies on black soldier fly larvae by Mshayisa et al. (2022), who also reported structural changes in insect protein flours as evidenced by enhanced amide and aliphatic C—H peaks in FT‐IR spectra. Similarly, studies on Maillard‐conjugated insect proteins confirmed the efficacy of FT‐IR in identifying protein‐polysaccharide interactions and tracking protein conformational changes (Hall et al. 2017).

From a food technology perspective, the observed changes suggest enhanced protein‐polysaccharide interactions and potential alterations in the techno‐functional behavior of the composite flours. Stronger amide signals may correlate with improved emulsifying and water‐binding capacities, while shifts in carbohydrate‐associated peaks may reflect changes in starch‐chitin interactions, which could influence dough rheology and textural properties in baked products. FT‐IR spectroscopy effectively demonstrated the integration of MWF into WF matrices, evidenced by intensified amide I and II bands and lipid‐ and carbohydrate‐related shifts. These spectral modifications confirm structural and compositional enhancements, supporting the potential of insect‐enriched composite flours in the development of high‐protein functional food products.

## 4 Conclusion

This study demonstrated that yellow mopane worm powder has considerable potential as a functional ingredient for improving the nutritional quality of foods formulated from nutrient‐poor raw materials. It was established that the addition of MWF can address deficiencies in certain amino acids and minerals, as significant increases in these nutrients were observed in flours with higher MWF levels (MW15% and MW20%). Hence, using this edible insect can address some of the FAO's requirements for amino acids required for human consumption. This study also showed that ingredients fortified with MWF would have enhanced shelf‐stability, which can be attributed to their low pH and A_W_ across all composite flours. An elevated oil‐binding capacity was observed in MW15% and MW20% flours, thereby highlighting their suitability in meat products. The increased foaming stability across all composite flours also indicates their suitability in baking applications, where soft, aerated textures are required. The pasting behavior of the composite flours indicated that incorporating insect flour into wheat flour at 5%, 10%, and 15% does not significantly affect the viscosities, namely the peak, setback, and final viscosities. The TGA and FT‐IR results revealed that the composite flours exhibit stable protein‐ and lipid‐associated bands with incorporation, thus increasing by confirming that MWF addition promotes the development of high‐protein foods and is suitable for oil‐absorption applications. Furthermore, DSC demonstrated that composite flours could serve a dual role in food applications, as they possess both endothermic and exothermic properties. Notwithstanding the promising nutritional, techno‐functional, and thermal properties of MWF‐enriched composite flours, this study has certain methodological limitations. The mechanisms underlying some functional properties, particularly oil‐binding and emulsification behavior, were inferred from amino acid composition and literature evidence, as protein surface hydrophobicity was not directly measured. Therefore, future studies should evaluate protein surface hydrophobicity to better elucidate its relationship with these functional properties. Further research is also required to assess the performance of yellow MWF in specific food applications, evaluate potential challenges in consumer acceptance and sensory appeal, and identify strategies to overcome food neophobia associated with edible insect consumption. Additionally, given the current lack of regulatory frameworks governing the use of mopane worms as food ingredients, future investigations should address food safety, allergenicity, and consumer protection considerations to support their wider utilization in food systems.

## Author Contributions

**Vusi Vincent Mshayisa:** writing – review and editing, writing – original draft, conceptualization, investigation, supervision, software, project administration, funding acquisition, formal analysis. **Tina Sinethemba Bebe:** conceptualization, writing – review and editing, writing – original draft, investigation, project administration, visualization, formal analysis, data curation, methodology. **Lusani Norah Vhangani:** writing – review and editing, supervision, validation. **Mpho Edward Mashau:** writing – review and editing, validation, supervision.

## Funding

This work was supported by Cape Peninsula University of Technology.

## Ethics Statement

The authors have nothing to report.

## Consent

The authors have nothing to report.

## Conflicts of Interest

The authors declare no conflicts of interest.

## Data Availability

The datasets generated and/or analyzed during the current study are available from the corresponding author upon reasonable request.
